# Reciprocality Between Estrogen Biology and Calcium Signaling in the Cardiovascular System

**DOI:** 10.3389/fendo.2020.568203

**Published:** 2020-09-29

**Authors:** Quang-Kim Tran

**Affiliations:** Department of Physiology and Pharmacology, College of Osteopathic Medicine, Des Moines University, Des Moines, IA, United States

**Keywords:** estrogen, G protein—coupled estrogen receptor, calcium, calmodulin, calmodulin-binding proteins, cardiomyocytes, vascular smooth muscle, endothelium

## Abstract

17β-Estradiol (E_2_) is the main estrogenic hormone in the body and exerts many cardiovascular protective effects. Via three receptors known to date, including estrogen receptors α (ERα) and β (ERβ) and the G protein-coupled estrogen receptor 1 (GPER, aka GPR30), E_2_ regulates numerous calcium-dependent activities in cardiovascular tissues. Nevertheless, effects of E_2_ and its receptors on components of the calcium signaling machinery (CSM), the underlying mechanisms, and the linked functional impact are only beginning to be elucidated. A picture is emerging of the reciprocality between estrogen biology and Ca^2+^ signaling. Therein, E_2_ and GPER, via both E_2_-dependent and E_2_-independent actions, moderate Ca^2+^-dependent activities; in turn, ERα and GPER are regulated by Ca^2+^ at the receptor level and downstream signaling via a feedforward loop. This article reviews current understanding of the effects of E_2_ and its receptors on the cardiovascular CSM and *vice versa* with a focus on mechanisms and combined functional impact. An overview of the main CSM components in cardiovascular tissues will be first provided, followed by a brief review of estrogen receptors and their Ca^2+^-dependent regulation. The effects of estrogenic agonists to stimulate acute Ca^2+^ signals will then be reviewed. Subsequently, E_2_-dependent and E_2_-independent effects of GPER on components of the Ca^2+^ signals triggered by other stimuli will be discussed. Finally, a case study will illustrate how the many mechanisms are coordinated to moderate Ca^2+^-dependent activities in the cardiovascular system.

## Main Components of the Calcium Signaling Machinery (CSM) in Cardiovascular Tissues

The CSM herein refers to proteins responsible for the generation or sequestration of intracellular Ca^2+^ signals and their transduction to target activities. In this section, key CSM components in cardiovascular tissues will be briefly described to facilitate review of the relevant effects and mechanisms of estrogenic agonists and receptors.

### Intracellular Ca^2+^ Stores, Release, and Uptake Mechanisms

#### Organelles Functioning as Intracellular Ca^2+^ Stores

The sarcoplasmic/endoplasmic reticulum (SR/ER) is the main Ca^2+^ store in cardiomyocytes, vascular smooth muscle cells (VSMCs) (1, 2), and endothelial cells (ECs), where the ER stores ~75% Ca^2+^ and mitochondria house ~25% (3). The Golgi (4, 5) and lysosomes have more recently been recognized as Ca^2+^ reservoirs (6, 7). Ca^2+^ reaches 5 × 10^−4^ M in the ER/SR and lysosomes and 1.3–2.5 × 10^−4^ M between the *trans*-Golgi and *cis*-Golgi (5, 8). The medial Golgi also releases Ca^2+^ in response to inositol-triphosphate receptor (IP_3_R) and ryanodine receptor (RyR) stimulation (9). Crosstalk between the ER/SR and other organelles affects their Ca^2+^ fluxes (10–14). In neonatal cardiomyocytes, beat-to-beat oscillations in mitochondrial and cytosolic Ca^2+^ occur in parallel (15), and mitochondrial uptake reduces cytosolic Ca^2+^ (16).

#### Mechanisms of Ca^2+^ Uptake Into Ca^2+^ Stores

SR/ER Ca^2+^-ATPases (SERCAs) are the key Ca^2+^ uptake mechanisms. For each ATP hydrolyzed, they pump 2 Ca^2+^ ions into the ER/SR in exchange for less than four H^+^ ions (17). SERCA2b is ubiquitously expressed. SERCA2a predominates in cardiomyocytes and is essential for cardiac development (18). SERCA3 is the predominant vascular isoform; its deletion causes smooth muscle relaxation abnormality (19, 20). SERCA3 has lower affinity for Ca^2+^ and is only active at high Ca^2+^ levels. Non-phosphorylated phospholamban interacts with SERCA1a, SERCA2a, and SERCA2b and reduces their Ca^2+^ affinity. Phosphorylation at Ser16 and Thr17 removes phospholamban–SERCA interaction, promoting SERCA activity (21, 22). Sarcolipin also binds SERCAs and reduces their Ca^2+^ affinity. Its deletion increases SR Ca^2+^ uptake (23).

The secretory pathway Ca^2+^ pump (SPCA) mediates Ca^2+^ uptake into the Golgi with nanomolar affinity for Ca^2+^. Unlike the SERCA, Ca^2+^ transport by SPCA is not associated with counter transport of H^+^. In the medial Golgi, both SERCA and SPCA participate in Ca^2+^ uptake (9).

Mitochondrial Ca^2+^ uptake is mediated by the voltage-dependent anion channel (VDAC) and the mitochondrial Ca^2+^ uniporter (MCU). VDACs are non-selective anion channels in the open state yet in the “closed” state permit influxes of cations such as K^+^, Na^+^, and Ca^2+^ into the mitochondria (24). VDAC isoforms participate equally in transporting Ca^2+^ triggered by IP_3_-producing agonists; however, VDAC1 selectively transports apoptotic Ca^2+^ signals (25). Myocardial VDAC2 regulates rhythmicity by influencing the spatial and temporal properties of cytoplasmic Ca^2+^ signals (26). The MCU constitutes a low-affinity yet selective Ca^2+^ channel pore as part of a mitochondrial Ca^2+^ uptake protein complex (MICU) and the essential MCU regulator (27, 28).

#### Mechanisms of Ca^2+^ Release From Ca^2+^ Stores

In IP_3_Rs, IP_3_ binds with IP_3_R2 > IP_3_R1 > IP_3_R3 affinity order (29) and cooperatively switches IP_3_R tetramers to an open conformation to form clusters and release Ca^2+^ (30, 31). IP_3_Rs regulate Ca^2+^ release from the ER/SR, Golgi apparatus, and nucleus (32). ER/SR Ca^2+^ release depletes ER Ca^2+^ and triggers store-operated Ca^2+^ entry (SOCE). IP_3_R2 predominates in the cardiomyocytes (33). In failing hearts, IP_3_R-mediated Ca^2+^ transients are enhanced, and mitochondrial Ca^2+^ uptake is reduced, which facilitates contraction and spontaneous action potentials that increase arrhythmogenicity (34). In VSMCs, all IP_3_Rs are expressed and are important for agonist-induced contraction (35). Endothelial IP_3_R1 is predominant in the brain (36), whereas IP_3_R2 and IP_3_R3 are abundant in the aorta and pulmonary arteries (37, 38).

*RyRs* (RyR1–RyR3) are the main SR Ca^2+^ release channels (39). *Regulation by cytosolic Ca*^2+^: In cardiomyocytes, RyR2 predominates (40) and is closed, activated, and inhibited, respectively, at Ca^2+^ <10^−7^ M, ~10^−7^-10^−5^ M, and >10^−3^ M (41). Entry via voltage-dependent Ca^2+^ channels (VDCCs) stimulates Ca^2+^-induced Ca^2+^ release (CICR) via RyR2, contributing to myocardial contraction. In VSMCs, RyR2 predominates in the aorta and pulmonary and cerebral arteries, while RyR3 is the only isoform in basilar arteries (42–44). CICR also contributes to VSMC contraction, but not as critically as in cardiomyocytes; indeed, skinned smooth muscle fiber bundles can contract at Ca^2+^ levels that do not activate RyRs (45). In ECs, RyR2 is on the ER and mitochondria (46); however, RyR agonists only cause a slow Ca^2+^ release that corresponds to a reduction in the IP_3_-sensitive Ca^2+^ pool (47, 48). *Regulation by SR Ca*^2+^ is important in cardiomyocytes. SR Ca^2+^ overload triggers spontaneous RyR2-mediated Ca^2+^ release, a phenomenon called store overload-induced Ca^2+^ release (SOICR) (49, 50). SOICR can cause delayed afterdepolarizations leading to tachycardias and is abolished by an E4872A mutation in the RyR2 gate (51).

### Ca^2+^ Entry

#### Store-Operated Ca^2+^ Entry (SOCE)

SOCE is a ubiquitous mechanism where Ca^2+^ store depletion triggers Ca^2+^ influx (52, 53). Proposed in the 1980s, SOCE was confirmed in the mid-2000s with the discoveries of the stromal interaction molecule 1 (STIM1) (54–56) and Orai Ca^2+^ channels (57–59). STIM1 resides mainly on the ER/SR membrane and has a luminal EF hand that houses a Ca^2+^-binding loop (60). In Ca^2+^-full ER/SR, the loop is in a closed conformation. Upon ER/SR Ca^2+^ depletion, Ca^2+^ leaving the loop promotes STIM1 oligomerization to interact with Orai1 channels and trigger Ca^2+^ entry (61–63). STIM1 also interacts with L-type Ca^2+^ channels (LTCCs) (64), maintains ER/SR structure (65–67), and is upregulated in atherosclerosis and hypertension (68–71). Myocardial SOCE is normally not prominent; however, STIM1 and SOCE are increased in heart failure (67, 72–76). In VSMCs, SOCE contributes significantly to contraction; α_1_AR-mediated contraction is reduced ~30% in SM-specific STIM1^−/−^ animals (77). In ECs, SOCE is the major Ca^2+^ entry and is required for many critical functions such as endothelial nitric oxide synthase (eNOS) activity and proliferation (78–82).

#### Voltage-Dependent Ca^2+^ Entry (VDCE)

Functional voltage-dependent Ca^2+^ channels (VDCCs) are the hallmark of tissue excitability and are present in cardiomyocytes and VSMCs, but not ECs. In cardiomyocytes, LTCCs are located mostly in transverse T tubules in apposition to RyR2s (83). Ca^2+^ entry via LTCCs triggers CICR via RyR2. In VSMCs, LTCCs also play a critical role in Ca^2+^ entry and contraction (84). The LTCC complex (85) consists of α_1_, α_2_, β, δ, and γ subunits. Four LTCC members are named according to their α1 pore-forming subunits: Ca_v_1.1, Ca_v_1.2, Ca_v_1.3, and Ca_v_1.4 (86). Ca_v_1.2 is predominant in cardiac and smooth muscles.

### Ca^2+^ Extrusion via the Plasma Membrane/Sarcolemma

The plasma membrane Ca^2+^-ATPases (PMCAs) prevail for Ca^2+^ extrusion in non-excitable tissues while the Na^+^-Ca^2+^ exchanger (NCX) is more important in excitable cells. SERCA2a, NCX, and PMCA sequester, respectively, ~70, 28, and 2% of cytosolic Ca^2+^ in cardiomyocytes (83) and 25, 25, and 50% in ECs (87).

#### Plasma Membrane Ca^2+^-ATPase

PMCAs extrude one Ca^2+^ ion for each ATP used and function as Ca^2+^-H^+^ exchangers (88–90). PMCAs are regulated by a Ca^2+^-dependent interaction with calmodulin (CaM). At low Ca^2+^, a C-terminal autoinhibitory domain binds to two cytosolic loops and inhibits pump activity. Increased Ca^2+^ promotes CaM–PMCA interaction, which removes inhibition and activates Ca^2+^ efflux (91, 92). PSD-95 promotes expression and distribution of PMCA4b via PDZ binding (93). PMCAs are inhibited by C-terminal tyrosine phosphorylation (94). Myocardial PMCAs play a little role under physiological conditions. However, expressions of PMCA1 and PMCA4 are reduced by up to 70 and 50%, respectively, in end-stage heart failure (95), and cardiac-specific overexpression of PMCA4b improved myocardial functions in ischemia–reperfusion injury and heart failure (96). PMCAs concentrate in the caveolae of VSMCs and ECs (97, 98). PMCA1 suppresses VSMC proliferation (99, 100), while PMCA4 mediates cell cycle (101, 102). In ECs, PMCA1b, and PMCA4b are predominant (87, 103, 104).

#### Na^+^-Ca^2+^ Exchanger

The NCX may function in two modes. In the *forward mode*, myocardial NCX1 balances LTCC-mediated Ca^2+^ entry and RyR-mediated Ca^2+^ release during cardiac excitation, extruding ~25% of the Ca^2+^ needed to activate myofilaments (105). NCX1 also predominates in VSMCs (106, 107). In ECs, NCX accounts for ~25% of Ca^2+^ removal (87). Endothelial NCX and PMCA dynamically adjust their Ca^2+^ extrusion rates to maintain sufficient efflux (104). In the *reverse mode*, upon myocardial depolarization, Na^+^ entry causes the NCX to transiently operate in this mode, promoting Ca^2+^ entry. This is much less efficient in triggering SR Ca^2+^ release compared to LTCC-mediated Ca^2+^ entry (108, 109). However, it primes the dyad to increase LTCC-mediated CICR (110). In VSMCs, reverse-mode NCX1 facilitates Ca^2+^ entry and mediates contraction, vascular tone, and blood pressure (111, 112). The reverse mode is not significant in ECs.

### Sex Differences in Ca^2+^ Signaling Proteins

Higher mRNA levels of Ca_v_1.2, RyR, and NCX, but not of phospholamban and SERCA2, have been observed in female than in male rat hearts (113). However, caffeine-induced Ca^2+^ release is lower in cardiomyocytes from female hearts (114). Ca_v_1.2 mRNA is higher in coronary smooth muscle from male than from female pigs (115). In smooth muscle cells (SMCs), expressions of ERα and ERβ, but not G protein-coupled estrogen receptor 1 (GPER), are higher in female than in male rats (116). These differences and the lower Ca_v_1.2 expression (115) may be responsible for less contraction of VSMCs from females (116). No studies have examined sex differences in Ca^2+^ handling proteins in ECs.

### Transduction of Ca^2+^ Signals—The Essential Role of Calmodulin (CaM)

While some Ca^2+^-dependent proteins are activated directly by Ca^2+^, many are activated by a complex between Ca^2+^ and CaM. CaM has two lobes linked by a flexible helix and can interact with ~300 target proteins (117, 118). Ca^2+^-free CaM binds or serves as structural subunits of ~15 proteins (119). However, each CaM lobe has two Ca^2+^-binding sites, and cooperative Ca^2+^ binding induces conformations that allow CaM to interact with many proteins, aided by the flexibility of the central helix (120, 121). Thus, CaM is the ubiquitous Ca^2+^ signal transducer. Activities of Ca^2+^/CaM-binding proteins depend on the Ca^2+^ signals, CaM availability, and properties of the interaction between Ca^2+^-CaM and the target proteins. Many of these factors are subject to estrogenic moderation.

Despite being required for activation of many Ca^2+^-dependent proteins, up to 50% of cellular CaM is engaged in inseparable interactions, leaving much less available for dynamic target binding (122). This generates an environment of limited CaM (123), as has been demonstrated in ECs (124), VSMCs (125), and cardiomyocytes (126). Consequently, competition for CaM generates a unique crosstalk among CaM-dependent proteins (124, 127), and factors that alter CaM level are predicted to have pervasive functional impact. It is noteworthy that virtually all CSM components interact with CaM and, in the context of reciprocality between estrogenic and Ca^2+^ signaling pathways, that ERα and GPER are both regulated by direct interactions with Ca^2+^-CaM.

## Estrogen Receptors and Their Calcium-Dependent Regulation

### Estrogen Receptor α (ERα)

ERα (128–130) is a nuclear receptor that, upon E2 binding (K_*d*_ ~ 10^−10^ M), assumes an active conformation to bind estrogen-responsive elements (EREs) in the promoters of target genes, modulating their transcription (131). Its N-terminus has a transcriptional activation function (AF-1) domain, a DNA-binding domain, and a hinge region; the C-terminus houses the ligand-binding domain and a second AF-2 domain. ERα is robustly expressed in the heart (132), VSMCs, and ECs (133–136).

ERα activities are strongly regulated by the Ca^2+^-dependent interaction with CaM. ERα binds CaM in a Ca^2+^-dependent fashion with a K_*d*_ of 1.6 ×10^−10^ M and an EC_50_(Ca^2+^) value of ~3 × 10^−7^ M (137). When ERα from Wistar rats' uteri is used, CaM decreases ERα-E_2_ binding but increases liganded ERα-ERE interaction (138, 139). A comparison of the CaM-bound/CaM-unbound ERα ratio in the cytosolic (unliganded) and nuclear (liganded) ERα pools isolated from MCF-7 cells suggests that E_2_ binding induces a conformation that favors ERα-CaM interaction (138). The CaM-binding domain was initially predicted to be a.a. 298–310 (137) but was later determined to be a.a. 298–317, with a.a. 248–317 required for maximal interaction (140). Further studies revealed that a.a. 287–311 is required to interact with both CaM lobes (141). CaM binding promotes ERα homodimerization that is critical for transcription activity (140, 142). With two lobes, each CaM binds two ERα molecules and thus stabilizes ERα dimerization (143). Notably, analogs of ERα17p (a.a. 295–311) that are unable to bind CaM downregulates ERα, stimulates ERα-dependent transcription, and enhances proliferation of MCF-7 cells, as does the wild-type ERα17p, indicating that this domain may also be involved in CaM-independent posttranslational regulation of ERα (144).

### Estrogen Receptor β (ERβ)

ERβ has ~96% and 55–58% sequence homology with ERα in the DNA- and ligand-binding domains, respectively (145, 146). ERβ binds E_2_ with a K_*d*_ of ~4–6 × 10^−10^ M. ERβ forms homodimers but more preferentially forms heterodimers with ERα, which bind E_2_ with a K_*d*_ of ~2 × 10^−9^ M and are transcriptionally active (147). ERβ is abundantly expressed in the vasculature (133–136). However, its expression and direct actions in the heart are controversial; cardiac manifestations in ERβ^−/−^ animals have been attributed to indirect effects from vascular changes (148). ERβ is not regulated by Ca^2+^ or CaM (149).

### GPER

GPER (150), aka GPR30, was cloned from various tissues in the 1990s (151–156). GPR30 is required for estrogenic activation of extracellular signal-related kinase (ERK)1/2 via transactivation of the epidermal growth factor receptor (EGFR) and release of the heparan-bound epidermal growth factor (EGF) (157, 158). It was shown to bind E_2_ in 2005 (159, 160), and the designation GPER was adopted by the International Union of Basic and Clinical Pharmacology in 2007 (161). A host of steroidal and non-steroidal agents and specific GPER agonists can activate GPER (150). GPER couples with Gα_s_ or Gα_i/o_. Supporting Gα_s_ coupling are data that (1) most membrane-bound [^35^S]GTPγ-S from cells overexpressing GPER and treated with E_2_ coimmunoprecipitate with Gα_s_ (159), (2) GPER is present in Gα_s_-pull-down fraction from GPER-expressing cells, and (3) E_2_ promotes GPER-dependent cyclic adenosine monophosphate (cAMP) production (162). Supporting GPER-Gα_i/o_ association are results that pertussis toxin prevents (1) E_2_-induced, GPER-mediated ERK1/2 phosphorylation in cells transfected with GPER (134, 157); (2) upregulation of c-fos in ERα/ERβ-negative, GPER-positive SKBr3 cells (163); and (3) E_2_-induced Ca^2+^ signals in ECs (164).

GPER is robustly expressed in cardiovascular tissues (133–136). In ECs, GPER mRNA is increased 8-fold by shear stress (154). GPER is localized on the ER/SR membrane (160) and responds to cell-permeable ligands (165). However, it also resides on the plasma membrane (166) and requires its C-terminal PDZ-binding motif to do so (167). The plasmalemmal GPER pool seems to constitutively undergo clathrin-dependent endocytosis and accumulate in the trans-Golgi network for ubiquitination in the proteasome without recycling to the plasma membrane, a process unaffected by agonist stimulation (168). Despite its predominant expression in the ER/SR, the sequence that drives GPER localization here has not been identified.

GPER is directly regulated by Ca^2+^-CaM complexes. In VSMCs and ECs, GPER coimmunoprecipitates with CaM in a constitutive association that is promoted by treatment with E_2_, G-1, or receptor-independent stimulation of Ca^2+^ entry (169, 170). GPER is the first G protein-coupled receptor (GPCR) shown to possess four CaM-binding sites on its respective four submembrane domains (SMDs) (169). Fluorescence resonance energy transfer (FRET) biosensors based on SMDs of GPER bind CaM with K_*d*_ from 0.4 to 136 × 10^−6^ M and affinity ranking SMD2 > SMD4 > SMD3 > SMD1. These interactions are Ca^2+^ dependent, with an EC_50_ (Ca^2+^) of 1.3 × 10^−7^-5 × 10^−6^ M, values within the physiological Ca^2+^ range (169). Due to technical challenges with purifying full-length GPCRs, the K_*CaM*_ for GPER as a holoreceptor is not available. The presence of four CaM-binding sites makes this task even more challenging and, in some way, not useful functionally. *Functionally*, mutations that reduce CaM binding but that do not perturb GPER-G_βγ_ preassociation drastically prevent GPER-mediated ERK1/2 phosphorylation (170).

## Stimulation of Calcium Signals by Estrogen and GPER Agonists

### Observations

In rat hearts, E_2_ (10^−12^-10^−8^ M) triggers ^45^Ca^2+^ uptake that is inhibited by LTCC antagonists (171). In VSMCs, GPER agonist G-1 triggers a slow-rising Ca^2+^ signal that is <2 × 10^−7^ M (172). In MCF-7 cells, E_2_ (10^−7^ M) induces Ca^2+^ store release and entry, yet only the former is required to activate mitogen-activated protein kinase (MAPK) (173). Interestingly, the ERα/ERβ antagonist ICI182,780 (10^−6^ M) also triggered Ca^2+^ signals in these cells. In ECs, E_2_ (10^−10^-10^−9^ M) triggers Ca^2+^ store release and entry, effects not affected by ERα/ERβ inhibitor tamoxifen (164, 174). The data with ICI182,780 and tamoxifen implicate a receptor other than ERα or ERβ in mediating the Ca^2+^ signal. Both reagents were later shown to be GPER agonists, triggering ERK1/2 phosphorylation only in cells expressing GPER (157, 159). Later studies confirmed Ca^2+^ signals stimulated by E_2_, GPER agonist G-1, and ICI182,780 in cells expressing GPER endogenously and absence of this effect in GPER^−/−^ cells (160, 175, 176).

### Mechanisms (Figure 1)

#### Direct E_2_-Ca_v_1.2 Interaction

E_2_ (10^−11^-10^−9^ M) potentiates *I*_Ca,L_ in neurons and HEK293 cells overexpressing the α1C subunit; nifedipine displaces membrane E_2_ binding; and E_2_'s effect is reduced by a dihydropyridine-insensitive LTCC mutant, indicating that E_2_ binds to the dihydropyridine-binding site (177). Intriguingly, E_2_ and the dihydropyridines exert opposite effects on *I*_Ca,L_.

**Figure 1:**
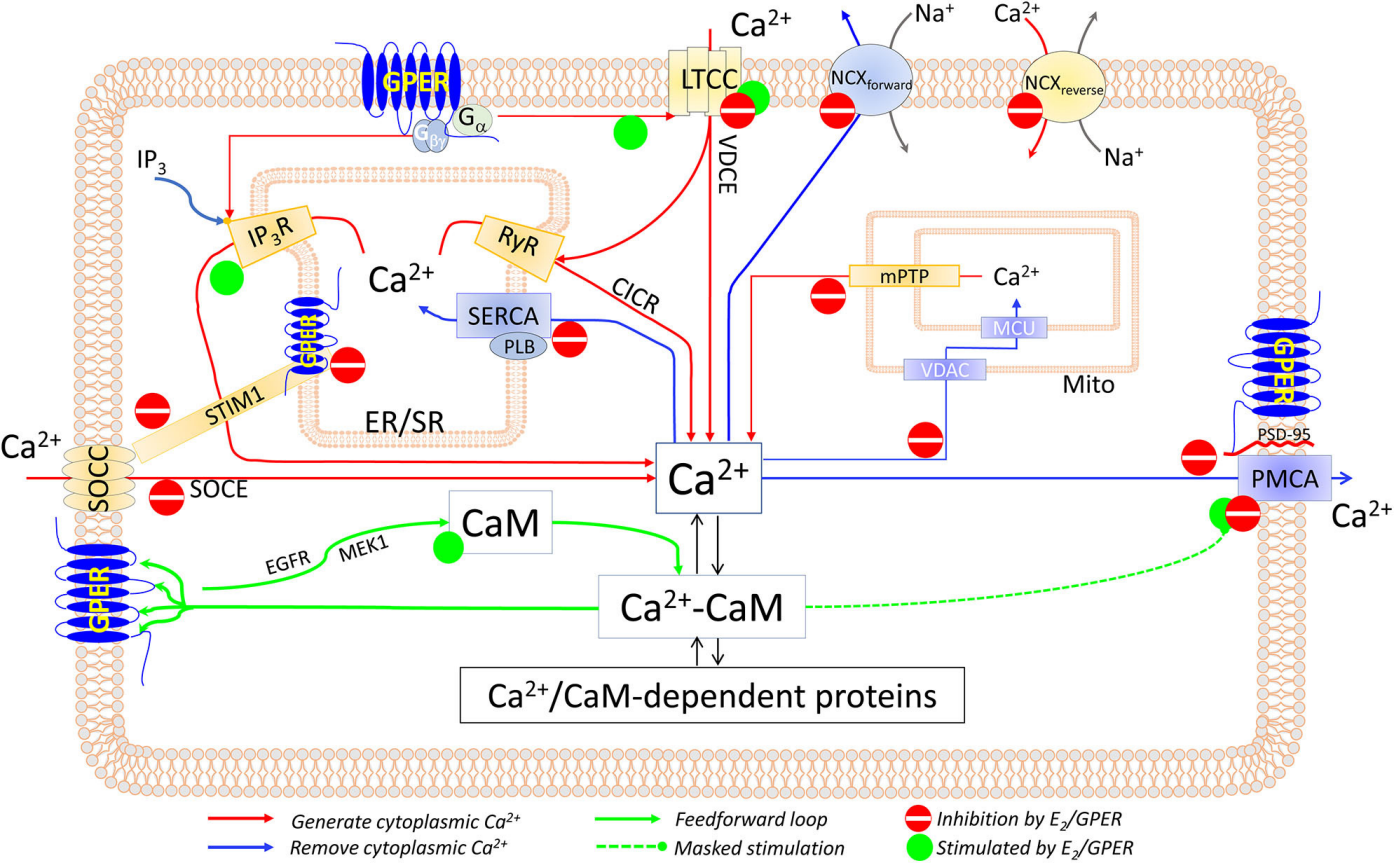
Components of the CSM that are affected by E_2_ and/or GPER in excitable and non-excitable cardiovascular tissues. See text for details.

#### Direct, Membrane-Delimited Activation of Ca^2+^ Channels by Gα Subunits

GPCR stimulation can trigger Ca^2+^ signals independently of the second messenger (178–180). GPER couples with Gα_s_ and Gα_i/o_, which can interact with LTCC (178, 181, 182) and trigger Ca^2+^ entry.

#### Release of G_βγ_ Subunit Upon GPER-Associated Gα_i_ Stimulation

G_βγ_ stimulates PLCβ (183–185) and activates IP_3_R1 (186), both of which trigger Ca^2+^ store depletion and SOCE. Consistently, E_2_-induced Ca^2+^ store release and entry in ECs are completely inhibited by pertussis toxin and PLCβ inhibitor U73122 (164). Also, HEK293 cells only produce a Ca^2+^ response to E_2_ when expressing HA-tagged GPER (162). Since (1) Ca^2+^ entry channels are located on the membrane and (2) G_βγ_ activates IP_3_Rs by interacting with the IP_3_-binding sites (186) on IP_3_Rs' cytosolic domains, both the membrane-delimited/Gα-mediated and G_βγ_-mediated mechanisms should only be operable by the plasmalemmal GPER pool. A distinguishing feature is that the former mechanism would not trigger SR/ER Ca^2+^ release in the absence of extracellular Ca^2+^, whereas the latter would. Based on this feature, data fitting the former are available from renal tubular cells (176); and data fitting the latter, from vascular ECs (164).

### Functional Impact

Do Ca^2+^ signals stimulated by estrogenic agonists activate Ca^2+^-dependent activities? When reported, the concentration of a Ca^2+^ signal allows for prediction of proteins that may or may not be affected by it. For example, E_2_ induces ER Ca^2+^ release signals of ~2 × 10^−7^ M and activates MAPK (173), because this Ca^2+^ level is sufficient for MAPK activity (187); indeed, Ca^2+^ chelation abolishes E_2_'s effect (173). Considering that GPER mediates the effect of E_2_ to trigger Ca^2+^ signals that activate MAPK, GPER activity can promote many downstream effects (163, 170, 188). In ECs, E_2_ (10^−9^-10^−6^ M) stimulates very small Ca^2+^ signals (<10^−7^ M) (174). One can predict that only proteins with very high Ca^2+^ sensitivity, for example, phosphorylated eNOS (170, 189, 190), would be activated by these signals. Whether a Ca^2+^ signal can produce a predicted effect also depends on other factors. For example, the Ca^2+^ signal of ~2 × 10^−7^ M triggered by G-1 in VSMCs (172) would be sufficient to activate myosin light-chain kinase (MLCK) and cause vasoconstriction, based on MLCK's properties (191). However, G-1 causes vasodilation (172, 192–194), likely by activating eNOS (170, 193, 195–198), inhibiting VSMC Ca^2+^ (199), and stimulating SMC K^+^ efflux (200).

## Calcium Entry Inhibition by Estrogenic Agonists and Estrogen Receptors

To a large extent, estrogenic regulation of Ca^2+^ signaling involves effects of estrogenic agonists and receptors on the Ca^2+^ signals triggered by other stimuli, via both E_2_-dependent and E_2_-independent mechanisms.

### Store-Operated Ca^2+^ Entry (Figure 1)

In VSMCs, E_2_ (10^−8^-10^−5^ M) inhibits norepinephrine- and phenylephrine-induced arterial constriction in the presence of extracellular Ca^2+^ but not that induced in Ca^2+^-free medium (201, 202). These effects may be attributed to inhibition of both VDCE and SOCE, as α_1_ adrenoceptor agonists can activate both (77). GPER-mediated inhibition of SOCE has been shown in ECs, where G-1 (10^−8^-10^−6^ M) suppresses SOCE induced by thapsigargin or bradykinin (203). Interestingly, the observations that in the absence of any treatment with agonists, thapsigargin-induced SOCE is increased by 80% in GPER-knockdown ECs and is reduced by 40% in GPER-overexpressing HEK293 cells implicate *E*_2_*-independent mechanisms* (203).

*How E*_2_*/GPER suppresses SOCE* seems to involve STIM1. G-1 treatment prevents thapsigargin-induced STIM1 puncta, indicating inhibition of STIM1's association with the Ca^2+^ channel; and Ser575/608/621Ala mutations of STIM1 reduce the inhibitory effect of G-1 (203). Consistently, E_2_ inhibits Ser575 STIM1 phosphorylation in bronchial epithelial cells, thus suppressing STIM1 mobility and SOCE (204). Our initial data also indicate that dynamic physical interaction between them contributes importantly to GPER's inhibition of SOCE (205).

### Voltage-Dependent Ca^2+^ Entry (Figure 1)

Electrically induced Ca^2+^ signals are increased in cardiomyocytes from ovariectomized (OVX) animals (206–208). Many lines of evidence indicate that GPER mediates the inhibitory effect of E_2_ on *I*_Ca,L_. These include inhibitory effects of E_2_ (1–3 × 10^−5^ M) and combined ERα/ERβ antagonists/GPER agonists (ICI182,780, tamoxifen, or raloxifene) on *I*_Ca,L_ in cardiomyocytes from both WT and ERα^−/−^/ERβ^−/−^ animals, as reviewed in (132). Similarly, in VSMCs, E_2_ inhibits electrically induced *I*_Ca,L_ (209, 210), and ERα/ERβ antagonists/GPER agonists tamoxifen and ICI164,384 inhibit high-K^+^-induced contraction (202). GPER agonist G-1 (10^−6^ M) inhibits nifedipine-sensitive Ca^2+^ spikes in LTCC-expressing A7R5 SMCs, an effect prevented by GPER antagonist G-15 (10^−6^ M) (199); these concentrations are specific for GPER (175, 211). Consistently, ERα knockout does not affect E_2_'s inhibition of KCl-induced ^45^Ca^2+^ uptake in VSMCs and vasorelaxation (212).

*How E*_2_ *inhibits electrically induced VDCE* is still unknown. Hypothetically, at high levels, E_2_ binding to the dihydropyridine-binding site on LTCC (177) may instead inhibit *I*_Ca,L_. As for prevention of β adrenoceptor (βAR)-mediated potentiation of VDCE, recent evidence suggests that GPER may be an intrinsic component of β_1_AR activation. Thus, G-1 inhibits isoproterenol-induced increases in left ventricle (LV) pressure, heart rate, ectopic contractions, *I*_Ca,L_, LTCC phosphorylation, and total myocardial Ca^2+^ signal, while the GPER inhibitor G-36 promotes ISO-induced Ca^2+^ signal and LTCC phosphorylation (213). Speculatively, GPER may do so in part by interacting with β_1_AR or with A kinase-anchoring protein 5, thus inhibiting cAMP production (167). These may represent some *E*_2_*-independent* effects of GPER. Studies in GPER-knockout tissues are needed to further clarify the mechanisms.

## Estrogenic Regulation of Cytoplasmic Calcium Removal Mechanisms

### SERCA Activity

Few studies, mostly in cardiac tissues, have examined the effects of E_2_ on *SERCA activity*, with somewhat conflicting results. E_2_ (1–30 × 10^−6^ M) does not affect the V_max_ of SR vesicle Ca^2+^ uptake in canine LV tissue (214). However, ovariectomy reduces the V_max_ but increases the Ca^2+^ sensitivity for SR Ca^2+^ uptake of rat LV homogenates or SR-enriched membrane fractions; *mechanistically*, these effects appear to be associated with reduced Thr17 phosphorylation of phospholamban and are restored by treatment with either E_2_ or progesterone (215) (Figure 1). How E_2_ and progesterone promote Thr17 phosphorylation of phospholamban is unknown, perhaps by inhibiting CaM kinase II (216), the enzyme that phosphorylates phospholamban (21). The effect of E_2_ on SERCA activity in VSMCs has not been examined.

### NCX Activity

As with SERCA activity, few studies have measured the effects of E_2_ on NCX activity. Na^+^-dependent ^45^Ca^2+^ uptake in rat LV myocytes is increased by ~3-fold after 60 days of ovariectomy, which is restored by replenishment with E_2_ (1.5 mg/60 days) (208). During myocardial ischemia, intracellular Na^+^ concentration is higher in male than in female cardiomyocytes and is associated with increased Ca^2+^ concentration as a result of increased NCX activity (217). These studies are consistent with an inhibitory effect of E_2_ on NCX activity in both the forward and reverse modes (Figure 1). However, the mechanisms of this inhibition are unclear.

### Mitochondrial Ca^2+^ Uptake

In the heart, diethylstilbestrol (0.9–1.8 × 10^−3^ M) inhibits mitochondrial ^45^Ca^2+^ uptake (218). Mitochondrial Ca^2+^ retention capacity (mCRC), a combination of mitochondrial Ca^2+^ uptake, total mitochondrial Ca^2+^-binding sites, and mitochondrial Ca^2+^ release mechanisms, is a determinant of the protective role of the mitochondria during cytoplasmic Ca^2+^ overload. E_2_ (4 × 10^−8^ M) increases myocardial mCRC following ischemia–reperfusion, an effect abolished by genetic deletion of GPER but not of ERα or ERβ; *mechanistically*, this effect seems to involve PKC-dependent, MAPK-dependent phosphorylation of glycogen synthase kinase (GSK)-3β, leading to inhibition of the mitochondrial permeability transition pore (219). Consistently, E_2_ (10^−8^ M) inhibits high Ca^2+^-induced cytochrome c release from myocardial mitochondria (220). In ECs, 48-h E_2_ (10^−8^ M) treatment inhibits mitochondrial Ca^2+^ uptake, an effect abolished by the ERα/ERβ antagonist ICI182,780 (10^−8^ M) (221). The mechanisms whereby E_2_ inhibits mitochondrial Ca^2+^ uptake are still unknown (Figure 1).

### PMCA Activity

Recent data show that GPER inhibits PMCA activity via both E_2_-dependent and E_2_-independent mechanisms (Figure 1). *E*_2_*-dependent mechanisms* are evidenced by the effects of G-1 (10^−8^-10^−6^ M) and E_2_ (1–5 × 10^−9^ M) to inhibit PMCA-mediated efflux in primary ECs without affecting PMCA expression levels and to promote PMCA phosphorylation at Tyr1176 (135, 170), which is known to inhibit pump activity (94). Notably, this phosphorylation masks the stimulatory effect of enhancing the PMCA–CaM interaction produced by 48-h E_2_ treatment (170). *E*_2_*-independent mechanisms* are indicated by the findings that (1) GPER constitutively interacts with PMCA4b via the anchoring action of PSD-95 at their C-terminal PDZ-binding motifs; (2) overexpression of GPER decreases PMCA activity; (3) GPER knockdown promotes PMCA activity; and (4) PSD-95 knockdown or truncation of the PDZ-binding motif on GPER releases GPER–PMCA association and promotes PMCA activity (135). *Functionally*, these mechanisms collectively prolong agonist-induced Ca^2+^ signal and enhance eNOS activity in ECs (135, 170, 203). Consistent with suppressed Ca^2+^ efflux, the Ca^2+^ signals stimulated by E_2_ and the GPER agonist G-1 in cells overexpressing GPER reported by various laboratories display much more prolonged plateau phases compared to Ca^2+^ signals in cells not overexpressing GPER or those stimulated by other agonists such as ATP or bradykinin (160, 162, 164, 175). GPER–PMCA4b interaction seems to be mutually influential, such that knockdown of PMCA decreases GPER-mediated ERK1/2 phosphorylation, while GPER knockdown does the opposite on PMCA activity (135).

## Estrogenic Regulation of Calcium Signal Transduction—The Calmodulin Network

Since CaM is the universal Ca^2+^ signal transducer for numerous proteins (117, 118), is insufficiently expressed for its targets (122, 125, 126), and is a source of competition among target proteins (124, 127), factors that regulate its expression and target interactions are predicted to have a pervasive impact. The effects of E_2_ on the CaM network have been examined in some detail in vascular ECs in recent studies (135, 169, 170). E_2_ treatment (1–5 × 10^−9^ M, 48 h) upregulates total CaM by around 7-fold and free Ca^2+^-CaM by ~15-fold in primary ECs. Data obtained using specific estrogen receptor agonists, gene silencing, and receptor overexpression indicate that GPER, but not ERα or ERβ, mediates this effect. Thus, the GPER agonist G-1 (10^−9^-10^−7^ M), but not the ERα agonist propyl pyrazole triol (PPT) (3 × 10^−10^-2 × 10^−7^ M) or the ERβ agonist diarylpropionitrile (DPN) (10^−10^-5 × 10^−8^ M), increases CaM expression; GPER knockdown reduces the effect of E_2_ to upregulate CaM; and E_2_ upregulates CaM in SKBR3 cells that express only GPER and not ERα or ERβ (170). Consistently, the ERα/ERβ antagonist/GPER agonist ICI182,780 dose-dependently upregulates CaM. *Mechanistically*, GPER exerts this action via the activities of EGFR and MAPK/ERK kinase 1 (MEK1). *Functionally*, E_2_ upregulates CaM and promotes the PMCA–CaM interaction; however, the predicted stimulatory effect on Ca^2+^ extrusion is masked by E_2_-induced inhibitory phosphorylation at Tyr1176 of PMCA (170); additionally, GPER exerts E_2_-dependent and E_2_-independent effects to inhibit PMCA (135). These collective actions prolong Ca^2+^ signals, promote Ca^2+^-CaM complex formation, and increase Ca^2+^-CaM associations with low- to high-affinity CaM network members, represented by GPER itself, ERα, and eNOS (170). Considering that CaM binding stabilizes ERα homodimers, these effects are expected to promote other genomic actions of E_2_ as well. Thus, a feedforward mechanism exists in which GPER mediates E_2_'s effects to increase CaM and inhibits Ca^2+^ efflux, prolonging cytoplasmic Ca^2+^ signals, and the resultant increases in Ca^2+^-CaM complexes in turn promote the activities of GPER itself and other CaM network members (170) (Figure 1).

## Estrogenic Moderation of Calcium-Dependent Activities

How do the various mechanisms discussed so far come together in regulating cardiovascular functions? An immediate challenge is how to reconcile the effects of estrogenic agonists to both trigger acute Ca^2+^ signals by themselves and inhibit otherwise stimulated Ca^2+^ signals. The Ca^2+^ signals triggered by estrogenic agonists in primary cardiovascular cells are generally of very low amplitude. Furthermore, as in experiments testing their effects on Ca^2+^ signals otherwise triggered, estrogenic agonists are present *in situ* with other stimuli whose Ca^2+^ signals they inhibit. Thus, for *mechanisms that generate cytoplasmic Ca*^2+^ *signals*, E_2_ and GPER exert ultimate inhibitory effects. For *cytoplasmic Ca*^2+^ *removal mechanisms*, estrogenic agonists and GPER also are inhibitory. For *Ca*^2+^ *signal transduction*, E_2_, via a feedforward at GPER, increases CaM expression and enhances linkage in the CaM-binding proteome.

**Figure 2:**
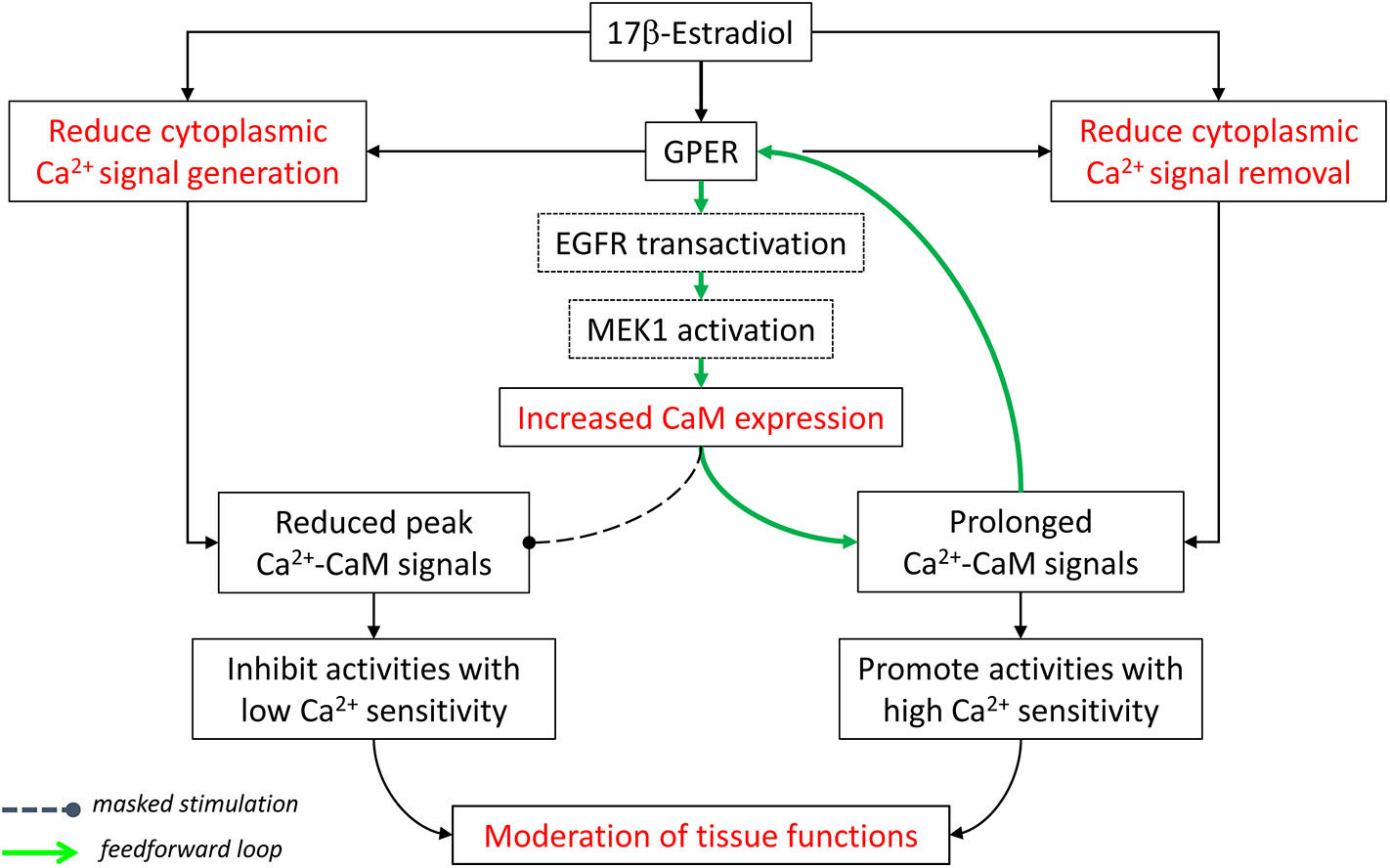
Moderation of cardiovascular functions by E_2_ and GPER via effects on Ca^2+^ signal generation, Ca^2+^ signal removal, and Ca^2+^ signal transduction. See text for details. Modified with permission from the author's previous publication (170).

All things considered, E_2_ and GPER, via both E_2_-dependent and E_2_-independent mechanisms, act to *moderate* Ca^2+^-dependent activities in the cardiovascular system. They “clamp” cytoplasmic Ca^2+^ signals by lowering peaks (inhibition of signal generation) and raising troughs (inhibition of signal removal), collectively confining tissues in a narrower yet more sustained operating range of Ca^2+^. Also, GPER-mediated increases in CaM expression and CaM network linkage improve Ca^2+^ signal transduction efficiency. Considering the Ca^2+^ sensitivity of Ca^2+^-dependent proteins in this context, one can predict that those with low Ca^2+^ sensitivity (requiring high Ca^2+^ for activation) are more likely to be affected by the inhibition of Ca^2+^ signal generation. On the other hand, proteins with high Ca^2+^ sensitivity (requiring low Ca^2+^ for activation) are more likely to be promoted by the inhibition of Ca^2+^ removal and less affected by the suppression of Ca^2+^ signal generation (Figure 2).

**Figure 3:**
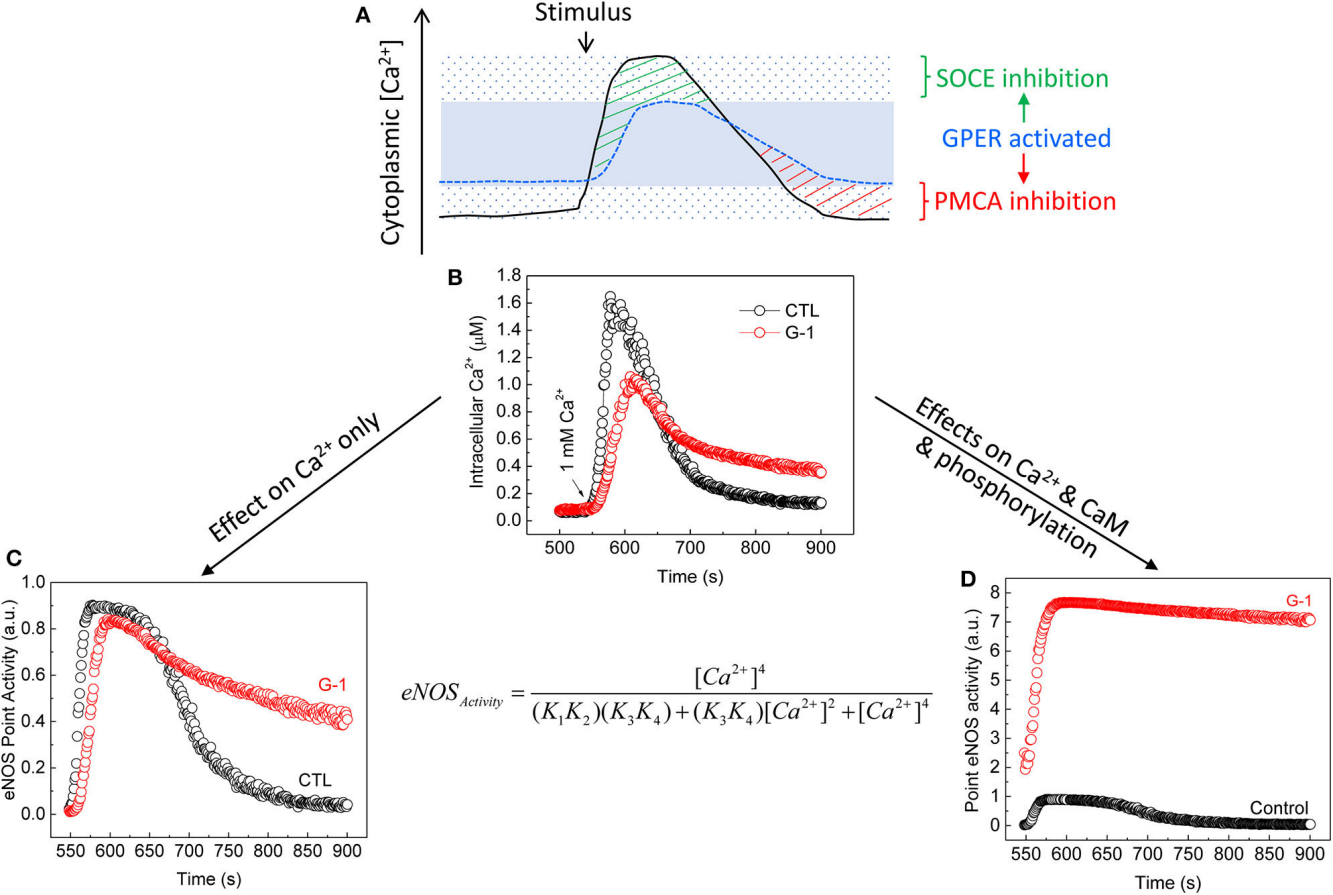
Moderation of Ca^2+^-dependent eNOS activity by GPER activation. **(A)** Cytoplasmic Ca^2+^ clamping by GPER activation in ECs (203). The solid line represents Ca^2+^ signals produced in response to agonist stimulation in the absence of GPER activation. The sparsely dotted area represents the range of cytoplasmic Ca^2+^ signals, in which peak and trough are seen due to maximal effects of Ca^2+^ entry and Ca^2+^ efflux. The stippled blue line represents Ca^2+^ signals produced in the presence of GPER and its activation. These signals are clamped in a narrower range (the blue area) due to inhibitory effects on both SOCE [green stripes (203)] and PMCA4b-mediated Ca^2+^ efflux [red stripes (135, 170)]. **(B)** Average time courses of cytoplasmic Ca^2+^ signals measured in primary ECs treated with bradykinin in the absence of extracellular Ca^2+^ followed by treatment with vehicle or G-1; total Ca^2+^ signals were triggered by re-addition of extracellular Ca^2+^ [arrow (203)]. **(C)** Calculated eNOS point activity corresponding to each Ca^2+^ value in **(B)** considering only changes in Ca^2+^ due to GPER activation using a verified sequential eNOS–CaM binding eNOS activation model [equation, where (*K*_1_, *K*_2_) and (*K*_3_, *K*_4_) are derived products of the binding constants of Ca^2+^ at the Ca^2+^-binding sites on the N and C lobes of CaM in binding to CaM and interaction of Ca^2+^-CaM and eNOS (189, 190). **(D)** Calculated eNOS point activity corresponding to each Ca^2+^ value measured in **(B)**, factoring in changes in Ca^2+^, CaM binding, and eNOS phosphorylation (170, 203). See details in text and (170, 203). Reproduced with permission from the author's previous publication (203).

This notion has been demonstrated experimentally via the case of eNOS, a Ca^2+^-dependent CaM-binding protein (222) with sub-nanomolar affinity for CaM (127). CaM interaction and subsequent activation of wild-type eNOS have high Ca^2+^ sensitivities, with respective EC_50_(Ca^2+^) values ~1.8 × 10^−7^ and 4 × 10^−7^ M (190). eNOS is also regulated by multisite phosphorylation (223). Notably, its bi-phosphorylation at Ser617 and Ser1179 promotes NO production by increasing the Ca^2+^ sensitivity for both CaM binding and enzyme activation, reducing their respective EC_50_ (Ca^2+^) values to ~0.7 × 10^−7^ and 1.3 × 10^−7^ M, thus rendering the synthase active at resting cytoplasmic Ca^2+^ (189). E_2_ and GPER (1) prolong endothelial cytoplasmic Ca^2+^ signal by inhibiting Ca^2+^ efflux (135, 170), (2) promote eNOS phosphorylation at Ser617 and Ser1179 (170, 198), (3) increase CaM expression and eNOS–CaM interaction (170), and (4) suppress endothelial SOCE (203). When we incorporate these effects into a verified sequential “CaM binding eNOS activation” model (189, 190), eNOS activity and NO accumulation are shown to substantially increase across the time course of bradykinin-induced Ca^2+^ signal in ECs by treatment with G-1 (203). Importantly, major contributions to this outcome include the increases in CaM binding, phosphorylation, Ca^2+^ sensitivity, and duration of Ca^2+^ signals due to Ca^2+^ efflux inhibition, but little or no effect of the inhibition of SOCE (203), due obviously to the synthase's high Ca^2+^ sensitivity (Figure 3). Thus, via multifaceted actions on components of the CSM, E_2_ and GPER moderate Ca^2+^-dependent activities by differentially affecting the continuum of Ca^2+^-dependent proteins based on their Ca^2+^ sensitivities for Ca^2+^ or Ca^2+^-CaM complexes.

Considering the two Ca^2+^-dependent estrogen receptors—ERα and GPER—how does the presence of one influence the effects of the other on Ca^2+^ signaling? A complex relationship is predicted to exist in which ERα transcriptional activities affect the expression of certain Ca^2+^ signaling proteins but are themselves influenced by the amplitudes and dynamics of Ca^2+^ signals limited by GPER activation and the availability of CaM that is promoted by GPER action (170). In turn, as CaM is limited in cells (122, 124, 126, 127), the high affinity binding of CaM by ERα and GPER further limits CaM availability and will influence CaM-dependent regulation of each other at the receptor level, a predictable outcome of the functional crosstalk via competition for limited CaM (124, 127). These relationships may represent but a small aspect of the reciprocality between estrogen and Ca^2+^ signaling.

## Conclusion and Future Perspectives

Reciprocality between estrogen signaling and Ca^2+^-dependent activities is becoming evident. *Considering the impact of estrogen and its receptors on Ca*^2+^ *signaling*, E_2_, and in many cases, GPER exert inhibitory effects on many components of the CSM in cardiovascular tissues, from Ca^2+^ store release and uptake (214, 215, 221) and Ca^2+^ entry (199, 201–210, 212, 213) to cytosolic Ca^2+^ removal mechanisms (135, 170, 208, 217–221). *Considering the impact of Ca*^2+^ *signaling on estrogen biology*, both ERα and GPER are strongly regulated by direct Ca^2+^-dependent interactions with CaM. These interactions serve to stabilize receptor dimerization and enhance subsequent transcriptional activities [the case of ERα (137, 138, 142, 143)] or promote receptor-mediated downstream signaling [the case of GPER (169, 170)]. Also, E_2_-induced MAPK activation has long been known to be dependent on the Ca^2+^ signal produced (173). Reciprocality between estrogen biology and Ca^2+^ signaling is further evidenced by the demonstration of *a feedforward mechanism*, in which E_2_, via GPER activation, upregulates total cellular CaM expression and free intracellular Ca^2+^-CaM concentration, which promotes functions of GPER and ERα and other classes of Ca^2+^-CaM-dependent proteins (170). The combination of these various actions is predicted to affect Ca^2+^-dependent functions depending on the affinity and Ca^2+^ sensitivities of the proteins involved, as exemplified by the case of eNOS (Figures 2, 3) (170, 203).

The moderating effects that estrogenic agonists and receptors exert on the CSM can explain many of their cardiovascular effects, such as preventing excessive cardiac contraction during sympathetic stress, limiting adverse outcomes related to Ca^2+^ overload, and reducing vascular tone. Nevertheless, the effects of E_2_ and estrogen receptors on many CSM components have not been examined. Additionally, many questions remain regarding mechanisms of the observed effects that estrogenic agonist and receptors produce on the CSM. For example, how do E_2_ and GPER inhibit *I*_Ca,L_? What are the mechanisms that position GPER as an intrinsic component of β_1_AR signaling in the myocardium? What are the mechanisms whereby E_2_ inhibits the activities of SERCA and NCX? What are the mechanisms whereby E_2_ inhibits mitochondrial Ca^2+^ uptake? Further studies are needed to answer these questions. Through many examples, however, it is clear that GPER produces both E_2_-dependent and E_2_-independent effects on the CSM. While the search is ongoing for approaches to apply specific estrogen receptor agonists to the prevention of cardiovascular disease, the therapeutic potential of E_2_-independent effects of GPER and other estrogen receptors is as yet an unexplored territory.

## Author Contributions

Q-KT conceived the ideas, generated the figures, and wrote the manuscript.

## Conflict of Interest

The author declares that the research was conducted in the absence of any commercial or financial relationships that could be construed as a potential conflict of interest.

## Abbreviations

AF domain: transcriptional activation function domain
CaM: calmodulin
Ca^2+^-CaM: Ca^2+^-bound calmodulin
cAMP: cyclic adenosine monophosphate
CICR: Ca^2+^-induced Ca^2+^ release
CRAC: Ca^2+^ release-activated channels
CSM: Ca^2+^ signaling machinery
E_2_: 17β-estradiol
ECs: endothelial cells
EGFR: epidermal growth factor receptor
eNOS: endothelial nitric oxide synthase
ERβ: estrogen receptor β
ERα: estrogen receptor α
ERK1/2: extracellular signal-related kinases 1 and 2
FRET: fluorescence resonance energy transfer
GPER: G protein-coupled estrogen receptor 1
GPR30: G protein-coupled estrogen receptor 1
HEK293 cells: human embryonic kidney 293 cells
*I*_Ca, L_: L-type Ca^2+^ channel current
IP_3_Rs: inositol-trisphosphate receptors
LTCC: L-type Ca^2+^ channels
LV: left ventricle
MAPK: mitogen-activated protein kinases
mCRC: mitochondrial Ca^2+^ retention capacity
MCU: mitochondrial Ca^2+^ uniporter
MEK1: MAP (mitogen-activated protein) kinase/ERK (extracellular signal-regulated kinase) kinase 1
mPTP: mitochondrial permeability transition pore
NCX: Na^+^-Ca^2+^ exchanger
OVX: ovariectomy/ovariectomized
PDZ: PSD-95/Dlg/ZO
PKC: protein kinase C
PLCβ: phospholipase C-β
PMCA: plasma membrane Ca^2+^-ATPase
PSD-95: post-synaptic density protein 95
RMP: resting membrane potential
RyRs: ryanodine receptors
SCPA: secretory pathway Ca^2+^ pump
SERCA: sarcoplasmic/endoplasmic reticulum-ATPase
SMD: submembrane domains of G protein-coupled receptors
SOCE: store-operated Ca^2+^ entry
SOICR: store overload-induced Ca^2+^ release
SR/ER: sarco-endoplasmic reticulum
STIM1: stromal interaction molecule 1
VDAC: voltage-dependent anion channel
VDCC: voltage-dependent Ca^2+^ channels
VDCE: voltage-dependent Ca^2+^ entry
VSMCs: vascular smooth muscle cells.
